# Supplementary material for: Are carbon emissions trading and green financial instruments synergistic? -Comprehensive quantitative research based on content analysis
Source: PLoS One. 2024 Mar 7;19(3):e0298601. doi: 10.1371/journal.pone.0298601 (PMC10919601; doi:10.1371/journal.pone.0298601)
Supplement: S1 Data — (ZIP) [file pone.0298601.s001.zip › Data/地级市Z_F工作报告.zip!/┴╔─■/╡ñ╢1⁄2╩╨/└┤╫╘╣1⁄2╓┌║┼í╛┬φ┐╦╩2╛▌═°í┐_2017.docx]

各位代表：

　　现在，我代表市人民政府向大会报告工作，请予审议，并请市政协委员和其他列席人员提出意见和建议。

　　一、2016年工作回顾与总结

　　过去一年，是丹东“十三五”规划的开局之年，是推进供给侧结构性改革的攻坚之年，也是振兴东北老工业基地继往开来的关键之年。面对异常严峻的宏观经济形势，我们在省委、省政府和市委的坚强领导下，深入学习贯彻党的十八大和十八届三中、四中、五中、六中全会精神，全面落实创新、协调、绿色、开放和共享发展理念，抢抓东北新一轮振兴和加快沿边开发开放机遇，实现了经济平稳运行。预计实现地区生产总值930亿元，下降5%，三次产业增加值比重为14:39.5:46.5;规模以上工业增加值68亿元，下降15%;固定资产投资280亿元，下降52%;社会消费品零售总额530亿元，增长5%;一般公共预算收入68.5亿元，增长3.1%;税收收入49.6亿元，增长1.4%;外贸出口总额24亿美元，下降9.7%;城镇常住居民人均可支配收入26084元，增长5.5%，农村常住居民人均可支配收入13370元，增长7%。

　　(一)供给侧结构性改革和产业转型升级加快推进

　　特色高效农业稳步实施。粮食生产和“菜篮子”供给平稳，粮食产量突破百万吨，实现“六连丰”。肉蛋奶产量达39.1万吨。调减玉米种植面积10.8万亩。新增投资亿元以上农业产业化项目8个、年销售收入亿元以上农业产业化龙头企业3户、规模农产品加工企业5户。无公害、绿色农产品生产认证达到77%，农作物耕种收综合机械化水平达到75%。新建畜禽标准化规模养殖场6个，实施畜禽标准化养殖项目3个。新增软枣猕猴桃、林下中药材等林地经济示范基地10个。落实玉米种植等农业补贴资金5亿元。“互联网+现代农业”行动和国家农业信息进村入户工程顺利推进。6个省级以上出口示范区带动作用明显，预计全年农产品出口额为6.8亿美元，居全省第2位。东港被评为“全国农产品质量安全示范县”，宽甸被评为“全国农村一二三产业融合发展示范县”。

　　依靠科技创新推动工业转型升级。《中国制造2025丹东实施方案》得到有效落实，出台了《科技创新驱动发展实施方案》《关于促进中小企业“专精特新”发展实施方案》《发展民营经济工作落实方案》等文件，推动了成长性好、发展潜力大、业绩良好、行业地位突出的“小巨人”企业快速成长。13户科技型企业被认定为国家高新技术企业。思凯公司入选国家互联网与工业融合创新试点企业，东方测控、华日理学、奥龙射线等企业成为省级智能制造示范企业。黄海新能源汽车产业基地项目竣工投产，获得国家补贴资金5.8亿元。全年实施智能制造项目45个，科技开发项目150个，其中达到国内领先以上项目61个，填补国内空白项目23个。特色产业集群建设取得新进展，丹东获得“中国满药之都”称号，东港获得“中国运动户外服装名城”称号。黄海大市场获得“国家小型微型企业创业创新基地”称号。全年化解煤炭产能42万吨、生铁产能30万吨。

　　现代服务业发展势头良好。旅游业在服务业中的带动作用进一步加强，重点旅游项目建设取得突破，总投资6.5亿元的北黄海温泉度假村和总投资10.4亿元的宗裕城金汤水世界两个温泉旅游综合体项目去年4月和7月相继投入使用，共接待游客100万人次，总收入超过1.5亿元;总投资20亿元的蒲石河森林公园、总投资10亿元的爱河生态公园、总投资2亿元的凤上线铁路综合旅游等一批项目实现开工;凤凰山景区成为我市首个旅游综合收入超亿元景区。边境旅游集聚效应进一步放大，完成了《丹东国家边境旅游试验区建设实施方案》申报和《丹东国家跨境旅游合作区建设实施方案》编制工作;开通了国内唯一一条赴朝开放线路全境游及赴新义州登岸游项目，全年赴朝旅游6.2万人次，创历史新高。区域旅游合作范围不断扩大，旅游市场专项整治成效明显，旅游基础设施进一步完善，与千客公司携手打造了“我爱丹东”系列旅游纪念品。金融业稳步发展，银行机构本外币贷款余额1121亿元，增长8 %，全年实现各类融资225亿元;设立了5亿元的政府产业投资引导基金，引进了我市首家3.5亿元的创业投资基金——国合金服;仁达食品、达荣科技2家企业在新三板挂牌;在东港启动了农村承包土地经营权和农民住房财产权抵押贷款试点。电子商务业迅速发展，建设电子商务产业园6个、电子商务平台40余个，东港成为国家电子商务进农村综合示范县，全市电子商务年交易额同比增长233%。夜经济日趋繁荣，发展安东老街等“夜经济”特色街27条、聚集区9个。房地产市场平稳发展，通过出台政策、召开房交会、赴外促销等措施，消化房地产库存210万平方米。

　　重点领域改革有序推进。启动事业单位分类改革和城建、交通、水务、旅游四大市属企业集团组建工作。积极落实“营改增”和资源税税制改革。通过合理安排预算、争取置换债券等方式，积极偿还政府债务，仅使用置换债券就节约利息支出2.6亿元。组建市土地经营权流转市场及监管中心，如期完成省政府下达的230万亩农村土地承包经营权确权任务。13个国有林场改革正式启动。不动产登记工作全面铺开。“双随机一公开”监管制度开始实施。县级公立医院改革取得实质性进展。

　　(二)招商和项目工作成效明显

　　重点项目建设进一步加快。采取集中开工、签订责任书、包扶重点项目、调度督查进展情况等措施全力推进项目建设，全市开复工投资亿元以上项目186个，计划投资总额1080亿元。其中新开工项目57个，计划投资总额249亿元。省政府重点调度的156个项目全部实现开复工。总投资17.8亿元的三湾水利枢纽项目并网发电，总投资5.2亿元的德丰时代建材广场投入使用，总投资20亿元的生物基尼龙56一期、总投资10亿元的中科钟表珠宝产业园等一批项目竣工。总投资10亿元的摩利特智能电气设备、总投资7.8亿元的广耀王老吉饮料、总投资4.8亿元的联诚高纯氢氧化镁等一批项目开始设备安装。总投资6.4亿元的大羽包装高速食品饮料罐、总投资3亿元的约翰森汽车配件等一批新材料、新能源、先进装备制造项目开工建设。

　　招商引资力度进一步加大。采取市县乡层层签订责任状、“四大班子”和市直部门落实指标任务等措施，全力推进招商引资工作。市领导率队赴北京、天津、山东、深圳等地开展精准招商，葵花生物科技、国泰新能源、辽旭汽车自动化检具等一批大项目签约落地，全年引进内资到位资金122亿元。有针对性地进行外资招商，洽谈了华润集团天然气等一批外资项目，接待了香港联泰集团等境外团组24个、客商100余名，储备外资项目32个，总投资7亿美元。

　　营商环境进一步优化。加大简政放权、转变政府职能力度，出台了《丹东市简化优化公共服务流程 方便基层群众办事创业工作方案》《市政府公共服务事项目录》等文件，共取消、下放、调整审批事项708项。市行政审批局挂牌成立。开展了软环境集中整治活动，整改审批、营商、行政执法等方面的问题68个。通过强化创业培训、建设创业基地、发放小额担保贷款等方式，加大对小微企业和创业个人扶持力度。“三证合一”成果显著，率先在全省开展“五证合一”“一证一码”试点，完成个转企805户。积极支持众创空间发展，科苑创客空间入驻创业企业41户。

　　政策红利进一步落实。《中共中央国务院关于全面振兴东北地区等老工业基地的若干意见》《国务院关于深入推进实施新一轮东北振兴战略 加快推动东北地区经济企稳向好若干重要举措的意见》《辽宁省人民政府关于支持丹东加快发展若干政策措施》等利好政策相继出台，为丹东振奋了精神，提供了机遇。我市相继出台了《任务分解方案》和《三年滚动计划》等落实文件，包装了400多个项目，估算投资4000多亿元;包装储备PPP项目26个，估算投资310亿元，丹东垃圾处理场、大孤山污水处理厂等公益项目得以推进。

　　(三)对外开放水平持续提升

　　“以港兴市”战略加快实施。制定出台了《关于加快丹东港口和临港产业发展的意见》，预计丹东港货物吞吐量达到1.58亿吨，增长5.1%;集装箱吞吐量达到192万标箱，增长4.9%。港口建设水平不断提高，港口泊位数量达到48个，在东北腹地设立陆港达到30多个。大东港疏港高速公路项目路基工程完工。临港产业快速发展，油脂加工一、二期和稻谷加工项目建成投产;海洋工程船舶制造形成规模，“致远”号全尺寸纪念舰主体建造完毕;进口木材贸易加工园海关监管区建成投入使用。

　　新区建设稳步推进。合作区成为中国开发区协会边境合作区分会首任会长单位，汽车及零部件、现代农业、仪器仪表等产业集群进一步壮大，挂牌成立了国家级科技企业孵化器;交通、商业、餐饮、医疗等配套设施不断完善，实验小学新区分校开工建设。高新区科技创新能力进一步提升，中科院育成中心运行良好，基础设施和配套服务项目加快实施。

　　对外开放平台作用得到发挥。《辽宁丹东重点开发开放试验区建设实施方案》重新修改后上报省政府，同步启动了功能区建设和综合保税物流园区申报工作。丹东机场临时开放获批，准备开通丹东-平壤国际航线。国门湾中朝边民互市贸易区投入试运营。出境加工业务试点带动了域内外53家企业开展业务。

　　外贸出口逆势而为。努力克服半岛形势紧张给我市出口带来的严重影响，采取有效措施帮扶外贸出口企业，确保民生领域对朝贸易正常进行。积极开拓欧美、非洲等新兴出口市场。天富贸易等10家外贸综合服务企业获得省商务厅重点扶持。国门云购、网贸国际等4个跨境电商平台上线运营，累计带动出口1亿多美元。全市外贸出口增幅位居全省第3位。

　　(四)民生福祉不断改善

　　社会保障水平持续提升。城镇新增就业2.3万人，城镇登记失业率控制在4.5%以内。通过加大扩面征缴、压缩一般性支出、减持国有股权等措施，保证了养老金及时足额发放。城镇职工养老金、最低工资标准，城市、农村低保标准，农村五保户供养标准，城镇居民基本医疗保险、新农合补助标准均有所提高。重度残疾人护理补贴和贫困残疾人生活补贴得到落实。

　　扶贫攻坚成效明显。全市139个党政机关、136户规模企业与124个贫困村进行了对接帮扶，各级财政共投入扶贫专项资金近5000万元，实施贫困村基础设施项目15个、进村入户产业扶贫项目566个，完成了“全市42个贫困村‘销号’、1.5万贫困人口脱贫，东港市和振安区全部脱贫”的任务。

　　城市基础设施不断完善。六道沟转盘实现环岛双向通行，解决了困扰市民5年之久的交通梗阻问题。金山高架桥建成通车。改造后的站前广场实现了营运车辆、私家车和行人的科学分流。黄海大街、振八街、兴五路等30条道路大修、改造完工。四号干线项目爱民街至宝山大街贯通工程开工建设。新增新能源公交车266辆,新增城区停车位1300多个。花园河治理和青年湖公园景观提升工程竣工。市区供热、供气、供水管网改造有序开展，新增城市热电集中供热联网面积750万平方米，联网总面积达到2690万平方米。

　　安居工程全面铺开。全市新开工建设棚改房9826套，货币化安置6519套，基本建成6826套。爱民沟、铁矿沟、毛绢厂、振山北等棚户区改造工作启动。对22个老旧小区实施了综合整治。

　　(五)社会事业和社会管理有序开展

　　教育、文化、卫生等工作得到加强。全市认定76所普惠性民办幼儿园，8000余名幼儿受益;凤城顺利通过国家义务教育发展基本均衡县评估验收;金汤小学、六纬路小学、第四幼儿园等改扩建项目顺利实施。公共文化服务水平进一步提升，“打造具有地域特色的传统节日文化活动”列入第二批国家公共文化服务体系示范项目。抗美援朝纪念馆改扩建工程全面推进，主体结构封顶。完成“致远”舰水下考古调查。开展食品安全城市创建工作和农村食品安全“扫雷”行动，小作坊、小饭桌、网络订餐得到有效监管。基本药物制度深入实施，新农合制度进一步巩固，分级诊疗、社会办医等工作取得新进展，全面两孩政策得到落实;全市病媒生物防控通过专家现场考核，综合评价达到国家标准。新建示范性城市社区全民健身中心10个;丹东籍运动员郑姝音里约奥运夺冠，为家乡争得荣誉。农村村委会换届工作在全省率先完成。我市第六次全国农业普查工作进展顺利。连续八届被评为全省双拥模范城，连续七届被评为全国双拥模范城。民族地区进一步繁荣。

　　人居环境持续改善。生态建设全面推进，青山、碧水、蓝天工程进展顺利，耕地、森林、水资源保护力度不断加大，完成营造林面积约18万亩，闭坑矿植被恢复工作全部完成;清理整顿环保违规建设项目697个，取缔“十小”污染水环境企业14户，淘汰黄标车1.63万辆，拆除10吨以下燃煤锅炉116台，建立了秸秆禁烧和综合利用县乡村三级防控网络。我市环境质量长期位居全省前列。加强城市管理，市容环境进一步改善。深入推进宜居乡村、美丽乡村建设，共投入2亿元资金，改造农村危房3000余户，硬化村内道路800余公里，新增太阳能路灯6000余盏，全市行政村所在地实现路灯全覆盖。宽甸被确定为国家重点生态功能区，通过省级生态县验收。全国文明城市、国家卫生城市、国家环保模范城市创建工作正在积极推进。

　　边境地区安全稳定。加快推进立体化治安防控体系建设，实施“四警联勤”武装巡逻和边境军警联合巡逻新机制，在全省率先开展弃管小区“创安”工作。严打违法犯罪活动，开展了“缉毒会战”、打击“盗抢骗”等专项行动，重点实施了打侵财、整治江边治安乱象、整治城区交通乱象的“一打两治”行动，取得明显成效，城市平安指数进一步提升。“五位一体”联合管边控海机制得到完善，集中整治非法易物和到敏感水域非法捕捞等各类涉边热点敏感问题，拆除“三无”船舶47艘。加大安全生产专项整治力度，全市没有发生较大以上安全生产事故。积极引导和支持群众理性表达诉求、依法维护权益，一些信访积案得到有效化解。

　　(六)政府自身建设进一步加强

　　依法接受市人大及其常委会的监督，自觉接受市政协的民主监督，主动听取民主党派、工商联、无党派人士和人民团体的意见建议。全年办理人大代表建议256件、政协委员提案327件，办结率、满意率均为100%。“七五”普法全面启动，政务公开不断向基层延伸。依法规范行政权力运行，科学化民主化法治化决策水平不断提高。扎实开展“两学一做”学习教育，切实做好省委第三巡视组对我市反馈意见的整改工作，开展了“全面从严治党 共谋振兴发展”大讨论活动和机关作风专项整治活动，政府机关担当意识和行政效能极大提升。认真落实党风廉政建设主体责任，全面贯彻中央八项规定精神，公车改革任务顺利完成。行政监察和审计监督得到加强。

　　外事、侨务、台务、接待、老龄、档案、气象、公积金、地方志、防震减灾等工作也取得了新成绩。

　　各位代表，全市经济社会取得的成绩，是市委总揽全局、正确领导的结果，是市人大、市政协有效监督、全力支持的结果，是全市广大人民群众共同努力、不懈奋斗的结果。在此，我代表市政府，向辛勤工作在各个领域的同志们，向市人大代表、政协委员，向各民主党派、工商联、无党派人士和各人民团体，向中省直部门、驻丹解放军、武警官兵，表示衷心的感谢并致以崇高的敬意!

　　在总结工作成绩的同时，我们也清醒地认识到面临的困难和问题。一是经济下行压力依然没有得到根本缓解。二是大项目储备不足，新开工项目减少，导致固定资产投资大幅下降。三是改革创新力度不够大，企事业等领域改革推进不快，效果不突出。四是财政运行困难，养老金、政府债务等问题靠自身力量还难以解决。五是少数干部不敢担当、不愿担当问题仍然存在。这些问题我们将在下一步工作中采取切实有效措施加以解决。

　　二、2017年经济社会发展目标任务和重点工作

　　今年是党的十九大召开之年,是实施“十三五”规划承上启下之年，也是本届政府收官之年，随着全省政治生态的恢复，经济数据的做实，国家、省级层面政策红利的逐步释放，基础设施日臻完善转化的经济优势不断显现，“全面从严治党 共谋振兴发展”大讨论活动带来的思想统一、精神振奋、政通人和，都为我市经济加快振兴发展奠定了基础，积蓄了能量。因此，我们将坚定信心，抢抓机遇，树立强烈的发展意识和责任意识，以担当的工作作风和饱满的工作热情，确保在丹东新一轮振兴发展和全面建成小康社会的生动实践中取得更大成绩!

　　今年市政府工作的总体要求是：认真贯彻中央、省委经济工作会议精神，按照市委十二届三次全会的统一部署，坚持稳中求进的工作总基调，抢抓国家、省支持我市加快发展的重大机遇，持续推进供给侧结构性改革，全面实施“以港兴市、生态立市、创新驱动、开放兴边、全域旅游、优化提升”六大战略，积极开展“招商引资项目建设年”活动，着力振兴实体经济，努力提升经济发展质量和效益，加快推进丹东新一轮振兴发展。

　　全年经济社会发展的主要预期目标是：地区生产总值增长6.5%，规模以上工业增加值增长7%，固定资产投资增长12%，社会消费品零售总额增长9%，一般公共预算收入增长5%，外贸出口总额全省占比不低于5.1%，城乡居民收入增长和经济发展同步。为实现上述目标，将重点抓好以下八个方面的工作：

　　(一)坚持以“招商引资项目建设年”活动为抓手，加快推动全市振兴发展

　　大力开展项目建设。把招商引资和项目建设工作作为全市经济工作的生命线，下大力气抓好。年内重点实施投资亿元以上项目190个，计划投资总额1150亿元。其中新开工项目65个，计划投资总额310亿元。

　　新开工基础设施项目包括：总投资7.6亿元的大孤山至丹东城区天然气管道供气工程、总投资5.3亿元的鸭绿江界河整治工程、总投资5亿元的丹东垃圾处理场、总投资4.3亿元的山海大道工程(东港至赤榆段)等重点项目。

　　新开工工业项目包括：总投资9.5亿元的清图运杰新能源电动汽车电池、总投资5.2亿元的思凯电子智能计量表、总投资5.2亿元的凤城化工硼氨联产综合利用、总投资5亿元的高新区葵花生物科技、总投资5亿元的辽旭汽车自动化检具、总投资3亿元的百岁山矿泉水等重点项目。

　　新开工的服务业、农业等项目包括：总投资50亿元的国际会展城、总投资15亿元的大连雨丰食品基地、总投资12.5亿元的天悦城综合体、总投资10.5亿元的毛绢厂棚户区改造、总投资8亿元的鸭绿江文化旅游度假区、总投资7亿元的凤城绸厂棚户区改造等重点项目。

　　全力做好招商引资。在招商方式上，突出县(市)区主体招商责任，发挥工业园区主力军作用，将驻点招商、委托招商、专业招商与集群招商、产业链招商、存量土地和厂房招商有机结合, 尤其是开展好以商招商，支持现有“小巨人”企业吸引外来技术、资金和项目，逐步做大做强。在招商区域上，瞄准世界500强企业、大型央企和国内外知名企业，紧盯珠三角、长三角、京津冀等重点地区和日韩、欧美等重点国家,着力引进龙头企业和配套协作企业。在招商产业方向上，突出装备制造业、战略性新兴产业、现代服务业、优质高效农业招商。在招商项目落地上，采取目标责任制、重大项目代办制、领导包保责任制等措施，全力做好签约项目征地、规划、环评、生产要素保障等工作，全年预开工项目的80%在上半年实现开工建设。

　　持续优化营商环境。认真落实《辽宁省优化营商环境条例》，坚决消除各种隐性障碍和“潜规则”。深入推进“放管服”，开展新一轮行政职权的取消和下放工作，重点抓好下放权力的衔接。以市民服务中心搬入新办公场所为契机，尽力实现全部审批事项进厅，切实提高项目审批效率。全面推行“双随机一公开”监管机制，随机抽查事项100%覆盖市场监管执法事项。

　　(二)坚持“以港兴市”战略，进一步凸显港口对经济的拉动作用

　　推动港口转型升级。加快丹东港散货、汽滚、客滚和集装箱泊位等大型专业化泊位以及港口附属、配套设施建设。依托60平方公里的港区，大力开展第三方物流业务，拉长港口服务产业链，创新开展港口金融租赁、加工贸易、信息共享等业务。重点打造大东沟中心渔港冷链物流中心、老东北牧业现代有机农业产业集群、进口木材加工产业集群、综合保税区四大临港产业板块。

　　打造区域物流中心。不断强化丹东港的东北东部出海新通道作用，积极对接吉林省向南发展战略，支持丹东港集团积极参与吉林通化国际内陆港务区建设。加快发展港口中转功能，实施中粮集团丹东港200万吨玉米物流项目，努力建设“北粮南运”重要集散地，以及矿石进口、电煤分拨等大宗物资区域集散地。

　　加大保障力度。强化“以港兴市”领导小组总体协调作用，有效落实对港口的支持，推动丹东港集团与东港市、丹东新区产业发展衔接、融合，强化对港口融资、交通、供水、供电、供热等生产要素的配置和保障，积极构建“以港兴市、以港促产、以产带城、以城促港”的联动格局。

　　(三)坚持“优化提升”战略，深入推进供给侧结构性改革

　　积极调整农业发展方式。继续调减玉米种植面积，引导农民发展高效特色经济作物。大力实施农产品深加工，推进农业产业化发展，新增亿元以上农业产业化项目5个、省级农业产业化龙头企业3户。抓好4个特色现代农业示范区、6个省级以上农产品出口示范区建设，依托特色农产品，抓好农村电商建设。提高森林质量和林地综合利用水平，加快农村合作社发展。鼓励畜禽标准化规模养殖和水产健康生态养殖，积极发展优质高效特色农业、绿色农业、休闲农业，深入推进农业与二三产业融合，打造一批特色小镇。

　　进一步优化工业结构。盘活存量，优化增量，做大总量，重振丹东轻工业名城雄风。

　　——全力促进“小巨人”等企业成长壮大。制定出台促进“小巨人”企业加快发展实施意见。加强对重点企业运行监测，以及生产要素的供应、保障，积极帮助企业解决市场、资金、人才等实际问题。鼓励中小微企业与大企业建立协同合作关系，促进更多中小微企业上规模。加快思凯公司工业4.0智能表具自动化生产基地、东方测控公司4G无线专网等高新项目建设。

　　——积极推动传统产业优化升级。全力落实《中国制造2025丹东实施方案》，以推进工业化和信息化深度融合为主攻方向，大力促进仪器仪表、新能源汽车及高端零部件、轻型精密装备等高端制造业发展。着力培育互联网、大数据、云计算、物联网等新兴产业。积极发展消费品产业，落实国家、省消费品工业“增品种、提品质、创品牌”专项行动，加快满族医药及健康产品、纺织服装、农副食品、手表日化、工艺美术等特色产业发展。继续实施以煤炭、生铁为主的工业去产能。

　　——深入实施“创新驱动”战略。精心培育高新技术企业群体，全年组织认定高新技术企业20户以上。围绕重点产业，深化产学研合作，转移转化一批科技成果。推进创新创业公共服务平台建设和中小企业研发中心建设，提升企业自主创新能力。加快促进科技、金融深度融合，引导社会资本和金融资本支持企业创新。

　　做大做强现代服务业。积极打造具有丹东特色的高丽街，培育好安东老街和万达金街等商业街，进一步繁荣“夜经济”。乘势做大电子商务产业，适时成立电子商务协会，培养引进行业人才，推进电子商务产业园建设，打造国家级电商产业基地。加快农产品批发市场新建、改造升级步伐，以及农产品连锁零售终端体系建设。大力发展会展业。加快养老服务业发展，促进养老业与房地产销售有机融合，全年房地产去库存面积231万平方米。

　　加快推进基础性关键性领域改革。坚决破除体制机制障碍，优化空间布局、产业布局和资源配置，激发内生活力。稳步实施事业单位和国有企业改革，严格按照《丹东市分类推进事业单位改革中事业单位转制为企业的实施意见》要求进行改革;优化重组国有资产，促进城建、交通、水务、旅游四大市属企业集团下半年投入运营;持续做好驻丹央企“三供一业”主辅分离工作。深化商事制度改革，推行企业“五证合一”改革和个体工商户“两证合一”改革。做好农村土地承包经营权确权登记颁证工作。完成13个国有林场改革。全面推开城市公立医院改革，实施医疗、医保、医药联动工作机制和分级诊疗制度。

　　继续强化财政金融支撑保障作用。坚持打造“民生财政、发展财政、绩效财政”，坚决兜住民生底线，确保实现“保工资、保运转、保基本民生”目标。努力营造有利于实体经济发展的财政政策环境，培育持续型税源。加快推进财税体制改革，落实各项减税降费优惠政策，打好边境、民族、生态保护和“两抗”革命老区四张特色市情牌，积极向上争取补助资金。严密防范和化解财政运行风险。重点做好与开发银行、进出口银行、农业发展银行等政策性银行的合作。积极帮助企业用好出口信用保险等金融工具，防范收汇风险。全年实现融资210亿元。

　　(四)坚持以“全域旅游”战略为统领，推进“旅游+”产业融合发展

　　促进旅游产业的深度融合。通过旅游集团的组建和运营，做大做强旅游市场主体。开工建设抗美援朝红色旅游文化传承基地等项目，加快实施蒲石河森林公园、爱河生态公园等项目，力争凤上线旅游、东汤宝达温泉等项目投入运营。积极培育生态旅游、工业旅游、养生养老旅游、文化体育旅游等“旅游+”新业态。

　　持续打造鸭绿江国际旅游品牌。力争鸭绿江精品旅游带规划获批并实施。依托东北东部和北黄海新干线旅游联盟共同打造鸭绿江国际旅游品牌，加大对京津冀、长三角等重要客源市场开发促销力度。推进丹东国家边境旅游试验区创建和丹东国家跨境旅游合作区设立。协助、支持旅游企业做好赴朝旅游新产品开发。办好鸭绿江国际旅游节。

　　推动旅游景区提质升级。加大旅游宣传和旅游市场综合整治力度，完善旅游基础设施和公共服务设施。加快天桥沟国家5A级景区和青山沟、獐岛、大鹿岛国家4A级旅游景区创建，以及凤凰山申报国家5A级景区工作。推进辽宁(宽甸)生态旅游试验区建设和凤城、宽甸全域旅游试验区的创建工作，使丹东旅游从单一景区建设向全域旅游提升转变，由门票经济向产业经济转变。推动丹东旅游集散服务中心和赴朝旅游服务中心建设。

　　(五)坚持“开放兴边”战略，切实提升经济外向度

　　促进新区全面繁荣。充分发挥新区对合作区和高新区的统领作用，有序剥离合作区、高新区社会管理职能，下放市级国土、规划等管理权，让合作区、高新区轻装上阵，心无旁骛地开展招商引资和项目建设。加快新区交通、教育、医疗、商贸等功能配套和供水、供暖、道路等基础设施建设。大力发展合作区电子科技、不锈钢、钟表珠宝、仪器仪表、新材料及跨境电商等重点产业集群。推进高新区创建国家级高新区和军民融合产业园建设，努力打造全国防护纺织产业基地，充分发挥中科院丹东育成中心科技创新作用。努力把新区打造成推动丹东新一轮振兴发展的重要增长极。

　　抓好东北振兴和沿边开发开放各项政策的落实。全面贯彻落实上级各项利好政策，根据任务分解落实意见，谋划包装好项目，积极向上争取政策和项目资金，促进国家和省里的政策红利在我市落地生根、开花结果。争取《辽宁丹东重点开发开放试验区建设实施方案》获得国家批准，综合保税区申报工作取得实质性进展，国门湾边民互市贸易区正式营业。

　　扩大对外出口和投资。加大对重点外贸企业的包扶力度，推动外贸综合服务企业做大做强。积极拓宽出境加工试点范围。加强与军贸企业合作，有针对性地开拓东欧、中东、非洲等新兴出口市场。鼓励跨境电商发展。不断扩大高技术含量、高附加值和较强市场竞争力的产品出口比重。主动对接“一带一路”倡议和辽宁自贸实验区建设，强化与东北东部地区在港口、物流、旅游等方面的合作。支持曙光、丹东港、金丸、双增、华纳等企业开展海外投资。

　　(六)坚持民生为本，让人民群众有更多的获得感

　　继续实施精准扶贫。完善“一对一”帮扶工作机制，围绕“六个精准”，实行“五个一批”，确保42个贫困村脱贫“销号”和1.5万贫困人口脱贫。

　　继续提高就业和社会保障水平。推动社会保障由制度全覆盖到人群全覆盖。千方百计扩大就业，实现新增城镇就业2万人，城镇登记失业率控制在4.5%以内。切实做好城乡贫困人口救助工作。

　　继续做好棚改和农村危房改造。新增棚改房4642套、基本建成1000套。改造农村危房3807户。

　　继续完善城市基础设施。着力实施北部山区基础设施改造，新修、翻建铁矿路、果园沟路、仁忠街等北部山区道路，打通桃源东路和花园东路。继续开展集中供热联网和供水供气管网改造。实施九纬路、十纬路、临江后街等道路改造工程，新建十纬路地下人行通道。打通四号干线爱民街至宝山大街段。加快发展城市公交。科学设置交通信号灯、标线、标志、隔离栏等道路交通安全设施。新增城市停车位1000个。高标准实施城市绿化、美化、亮化工程。加快推进鸭绿江水源地上移工程。

　　继续抓好生态市创建和环境治理。大力实施“生态立市”战略，科学划定生态红线，持续开展蓝天、碧水、青山、沃土工程。积极推进大气污染防治工作，全部淘汰公交线路黄标车，更换成新能源汽车;拆除燃煤供暖锅炉41台;抓好秸秆焚烧污染防控和综合利用，确保城市环境空气质量优良天数率达到84.7%。加大水污染防治力度，确保全市河流水质优良比例达到87.5%，市区集中式饮用水源地水质达标率达到100%。切实做好土壤污染防治。加强城乡环境综合整治，以创建全国文明城市、国家卫生城市和国家环保模范城市为着力点，加快城乡污水处理厂、垃圾处理设施建设和运营，完成20个老旧住宅小区综合整治。

　　(七)坚持统筹协调，着力促进社会事业全面发展

　　提高教育基础设施建设水平。支持公办幼儿园建设，加快义务教育均衡发展进程，改造33所农村义务教育薄弱学校，积极推进丹东七中等学校建设，全市义务教育标准化学校建设比例达到80%以上，鼓励职业教育创新转型发展。

　　提高公共卫生服务水平。加强基层卫生服务能力，推进卫生计生信息管理市级平台建设和全民卫生应急能力建设，实现城乡居民基本医疗全覆盖。加快建立科学完善的食品药品安全治理体系，着力规范食品药品市场秩序，全面推进食品安全城市创建活动。

　　提高文化惠民服务水平。通过实施农家书屋、广播电视村村通、“流动图书馆”“流动大舞台”等方式，努力打通公共文化服务“最后一公里”。积极推进抗美援朝纪念馆改扩建工程，力争年内开馆。

　　提高公共体育服务水平。继续实施全民健身活动和“一区一品”群众体育品牌活动，统筹推进城乡多功能运动场建设。

　　提高维护国家安全和社会稳定水平。加大安全生产工作力度，抓好安全隐患排查整改。整合优化政府服务热线，畅通民意诉求渠道，提升信访工作水平，着力解决信访积案和历史遗留问题。认真做好国防和民兵预备役工作，巩固提高“双拥”工作成果，促进军民融合发展。积极推进立体化社会治安防控体系建设，加大管边控边工作力度，持续开展“三无”船舶专项整治行动，确保国家安全和边境地区和谐稳定。

　　(八)坚持勤政廉洁，不断增强政府执行力和公信力

　　全面依法行政。坚持法定职责必须为、法无授权不可为，依法履行政府职能。完善政府立法工作机制，落实法律顾问制度。认真做好人民调解和法律援助工作，全面实施“七五”普法规划。进一步加强行政执法监督工作，依法受理、审理行政复议案件。自觉接受人大依法监督、政协民主监督，认真听取各民主党派、人民团体和各界人士的意见建议，高度重视并认真办理好人大代表建议和政协委员提案。加大政务公开力度，主动接受社会监督、舆论监督。

　　深入转变作风。发扬“苦干、实干加巧干”的大梨树精神，巩固“全面从严治党 共谋振兴发展”大讨论活动和机关作风专项整治活动成果，严厉整治“不作为、慢作为、乱作为”问题，大兴“主动作为、勇于担当、敢做善成”风气。整合督察力量，实行督察考评合一，把抓落实放在政府工作更加突出的位置。以政务诚信示范引领全社会诚信建设，该负的责任再重也要扛在肩上，该干的事再难也要坚持做好，尤其是答应群众的事一定要言必信、行必果。

　　抓好廉洁建设。深入贯彻全面从严治党要求，不断完善防腐治腐的制度体系，积极落实“两个责任”，认真履行“一岗双责”，强化对公共资金、国有资产、国有资源和领导干部履行经济责任的审计监督，从严惩处各类腐败行为。自觉遵守《关于新形势下党内政治生活的若干准则》《中国共产党党内监督条例》，促进广大公务人员正确履职，保持忠诚、干净和担当，营造风清气正的振兴发展氛围。

　　各位代表，丹东新一轮全面振兴发展的宏伟蓝图已经绘就，全面建成小康社会决胜阶段的号角已经吹响，让我们在市委的正确领导下，在市人大和市政协的有力监督和支持下，进一步开拓创新，奋发进取，努力开创全市经济社会发展新局面，以优异的成绩迎接党的十九大胜利召开!
